# Personalized Respiratory Motion Modeling Incorporating Longitudinal Data through Two-stage Transfer Learning

**DOI:** 10.2174/0115734056325170250114210309

**Published:** 2025-01-21

**Authors:** Peizhi Chen, Xupeng Zou, Yifan Guo

**Affiliations:** 1 Xiamen University of Technology, College of Computer and Information Engineering, Xiamen, China

**Keywords:** Transfer learning, Longitudinal data, Respiratory motion modeling, adaptive radiotherapy, motion analysis, 4D-CT images

## Abstract

**Purpose:**

This study aims to develop an accurate image registration framework for personalized respiratory motion modeling.

**Methods:**

The proposed framework incorporates longitudinal data through a two-stage transfer learning approach. In the first stage, transfer learning is employed on longitudinal data collected from the same device. In the second stage, a personalized model is constructed using the transfer learning approach, reusing the model from the first stage. A novel cross-error function is introduced to guide the customized adaptation stage.

**Results:**

The experiments demonstrate the effectiveness of the proposed framework in respiratory motion modeling. Integrating longitudinal data allows for improved accuracy for personalized respiratory motion modeling.

**Conclusion:**

The study presents a novel approach that incorporates longitudinal data into a two-stage transfer learning process for personalized respiratory motion modeling. The framework demonstrates improved accuracy. The results highlight the potential of leveraging longitudinal data to provide personalized image registration solutions.

## 1 INTRODUCTION

Respiratory motion modeling is a crucial topic for analyzing chest images in medical imaging, as it aims to align anatomical structures in 4D-CT images throughout a complete respiratory cycle. The respiratory motion model is essential in adaptive radiotherapy [1]. In contrast to generic models, personalized respiratory motion modeling [2-5] aims to construct person-specific models. It is motivated by the desire to gain a better understanding and motion analysis in a specific individual.

Image registration [6] is the key to respiratory motion modeling. The existing methods can be categorized into two types: optimization-based and deep learning-based. Optimization-based methods, such as Hammer [7], SyN [8], and elastix [9], treat image registration as an optimization problem. However, these methods can be time-consuming, limiting their applicability in time-sensitive scenarios, such as image- guided radiation therapy [6]. On the other hand, deep learning-based approaches leverage neural networks, often with the assistance of Graphics Processing Units (GPUs), to learn the displacement between two images. In comparison to optimization-based methods, deep learning-based methods exhibit significantly faster registration times. Currently, unsupervised learning-based image registration methods are widely researched. VoxelMorph [10] stands out as a popular method that employs convolu- tional neural networks to learn the displacement between images in an unsupervised manner.

However, the existing methods for training networks often rely on population datasets without considering the valuable insights that can be gained from longitudinal data collected from the same devices. We propose a hypothesis that incorporating longitudinal data can improve respiratory motion modeling.

There are two existing methods for incorporating longitudinal data: federated learning and transfer learning. Federated learning involves utilizing distributed data from various institutions, allowing for a wider range of data to train the network. On the other hand, transfer learning can incorporate longitudinal data within the same institution. However, there is a concern regarding data privacy when directly using transfer learning methods.

In the paper, we propose a novel framework that incorporates longitudinal data into a two-stage transfer learning process, as is shown in Fig. (**1**). In the first stage, we leverage transfer learning to utilize the longitudinal data collected from the same device. Subsequently, when a new patient’s 4D-CT dataset becomes available, we con- struct a personalized model using transfer learning. By reusing the model from the first stage rather than the longitudinal data itself, we ensure the data protection of the longitudinal data.

Specifically, the paper introduces a novel two-stage transfer Learning method that utilizes longitudinal data with a cross-error function. The cross-error function provides a hierarchical solution for the knowledge learned from both historical and personal- ized data. Hence, we refer to this method as hierarchical transfer learning (HTL). We verify the effectiveness of this approach in respiratory motion modeling, making two contributions to the field. Firstly, we introduce a new model that integrates longitudinal data. Secondly, we propose a new error function that utilizes the knowledge learned from the longitudinal data.

## 2 METHOD

Our goal is to construct a learning-based personalized respiratory motion model with the aid of longitudinal data. To achieve this, we present a two-stage transfer learning (namely, hierarchical transfer learning) framework that implies longitudinal data using a cross-error function, as shown in Fig. (**2**).

### 2.1 Two-stage Transfer Learning

To develop a personalized respiratory motion model, we divide the process into two stages: device and personalized adaptation.

In the device adaptation stage, our goal is to construct a generic model that captures the characteristics of the device being used. This is important because longitudinal data are typically obtained using the same device, such as within a signal hospital. To achieve this, we apply a fine-tuning technique to an existing model using the longitudinal data.

Next, in the personalized adaptation stage, we acquire multiple 4D images using the same device, following the common practice in hospitals where consistent equipment is utilized. We refine the generic model to build a personalized model. The approach offers benefits in terms of data protection, as we only require the trained model instead of the longitudinal data. To explicitly incorporate the knowledge obtained from the generic model, we propose a novel error function.

### 2.2 Cross-error Loss Function

Transfer learning allows us to leverage the knowledge gained from the generic model and apply it to the personalized model. Since both models share the same domain and network framework, we expect the output of the personalized model to be similar to that of the generic model. To achieve this, we introduce a novel cross-error loss function.

In the personalized adaption stage, where 4D images are acquired using the same device, we fine-tune the genetic model to the personalized model. We design a novel error function named cross error *E_c_*. which is defined as:

**Table:** 

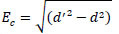

where *d^′^* and *d* are the displacement fields of a using the genetic and personalized models, respectively. The underlying idea behind this error function is that the insights gained from the generic model can be incorporated into the learning process of the personalized model through a registration result. Our goal is to guide the learning of the personalized adaptation stage in a way that preserves the capabilities obtained during the device adaptation stage.

### 2.3 Unsupervised Image Registration

We design an unsupervised learning-based image registration algorithm to verify the hierarchical transfer learning method. The input is a pair of images, and the output is their displacement field. We use a U-Net-like neural network to learn the displacement field without the requirement of supervised signals, like VoxelMorph [10, 2]. The neural network in both stages is the same.

### 2.4 Implementation

#### 2.4.1 Preprocessing

**Fig. (3):**
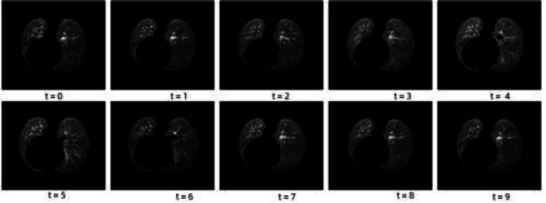
Examples of 4D CT images of the lung region.

In our paper, we focus on modeling the respiratory motion of the lung region. Since the experimental data consists of four-dimensional chest images, we perform a segmentation process to isolate the lung region (Fig. **3**). The segmentation is achieved using a threshold-based method.

Given that the Hounsfield Unit (HU) range of (-1000, 400) corresponds to the lung region, we only retain this specific region and normalize the HU values to fall within the range of (0, 1).

#### 2.4.2 Respiratory Motion Modeling

To develop a respiratory motion model, we utilize atlas-based image registration. In this approach, we select the end-expiration volume as the atlas image and registration is carried out for all volumes within the four dimensional image dataset. The goal is to align these volumes with the atlas image, resulting in a displacement field that is consistent with the atlas image.

## 3 RESULTS

### 3.1 Materials

The experimental materials are 4D-CT from the same device. We utilize the 4DLung [11-13] dataset, which comprises 4D-CT scans obtained at multiple time points from lung cancer patients. We demonstrate the effectiveness of HTL in the 4D- CT registration application. First, we use four pairs of four dimensional images from different patients to train the first transfer learning model and achieve a model that is fit for the device adaptation phase. Second, we select four-dimensional images from a specific patient to train the second transfer learning model. Finally, we verify the effectiveness of registration for the four-dimensional images from this patient-specific patient.

### 3.2 Baselines

several state-of-the-art methods will be compared The baseline methods include deep learning based methods: VoxelMorph [10], Dlung [14] and optimization-based methods: SyN [8] and Elastix [9]. We evaluate the methods using their public implementations.

### 3.3 Metric

To assess the accuracy of the dice, we evaluated its performance by measuring the overlap between registered and ground truth regions. The Dice score ranges from 0 to 1, with 1 indicating perfect overlap. Additionally, we analyzed the Jacobian matrix to examine the presence of pixel folding in the registered image. The Jacobian matrix was computed for each pixel, with negative values indicating folding. We counted the number of pixels with negative Jacobian values, and a lower number indicates fewer folding pixels and greater alignment accuracy.

### 3.4 Analytical Study

We performed ablation experiments and comparisons to evaluate the effectiveness of each component in the HTL model and to conduct an in-depth analysis.

#### 3.4.1 Effect of Hierarchical Transfer Learning

Through the ablation experiment, we investigated the impact of hierarchical transfer learning, which is designed to improve registration with longitudinal data. We compared the performance of our method with and without hierarchical transfer learn- ing, and the results are presented in Fig. (**4**). Our findings indicate that the proposed method with hierarchical transfer learning outperforms the one without it.

#### 3.4.2 Effect on Cross Error

The objective of the ablation experiment was to demonstrate the effectiveness of incorporating longitudinal data from the dataset with cross-error function. Our experiment results, depicted in Fig. (**5**), clearly indicate the effectiveness of the cross- error function in incorporating longitudinal data.

#### 3.4.3 Comparison of Training Mode

Our experiment focused on comparing training models using both present and longitudinal data. Patient-specifically, we compared the performance of models trained from scratch, *via* transfer learning, cross-error, and joint learning. The results are presented in Fig. (**6**), clearly indicating that incorporating longitudinal data produces the best performance.

### 3.5 Comparison with Baselines

To evaluate the performance of our method, we compared it with several baselines. Patient-specifically, we selected two deep learning-based methods (VoxelMorph and Dlung) and two traditional optimization methods (SyN and Elastix) as baselines.

#### 3.5.1 Dice Comparison

We compared the dice score of our method with that of the baseline and presented the results in Fig. (**7**). Our procedure, HTL, yielded superior results compared to the baseline.

#### 3.5.2 Jacobian Comparison

Our comparison involved measuring the Jacobian determinant of the different methods, with the results presented in Table **1**. The results show that our method outperforms others concerning the number of folding pixels.

## 4 DISCUSSION

Our paper proposes a novel framework for personalized respiratory motion modeling with longitudinal data. Specifically, we introduce hierarchical transfer learning with cross-error function to incorporate longitudinal data. Ablation studies verified the effectiveness of our contributions, highlighting the value of incorporating longitudinal data to improve the dice score by about 10%, with the similar performance achieved through joint learning algorithms. We further compared our proposed method with state-of-the-art methods, including deep learning- based and traditional optimization-based techniques. Our method achieved about 10% improvement in dice score compared to VoxelMorph and Dlung, with similar results to Dlung in the number of folding pixels. Compared to optimization based methods, our deep learning-based approach was faster.

Overall, our contributions demonstrated improved performance for image registration. Nevertheless, our study has the following limitations: the lack of training datasets. Future experiments will address these issues and provide further insights. The integration of longitudinal data appears to be an effective approach, but further research is needed.

## CONCLUSION

Our paper proposes a novel hierarchical transfer learning method with cross error to integrate longitudinal data from datasets, and we apply this approach to respiratory motion modeling. In our experiments, we demonstrate the effectiveness of hierarchical transfer learning and cross error, with our approach achieving about 10% improvement in dice score compared to state-of-the-art deep learning-based methods, and the number of folding pixels being like that of Dlung.

However, our work has limitations due to the lack of training datasets and appli- cations to different datasets. We aim to address these limitations through additional experiments. We find that incorporating longitudinal data is a valuable approach for improving model training, serving as a foundation for incremental learning.

## Figures and Tables

**Fig. (1):**
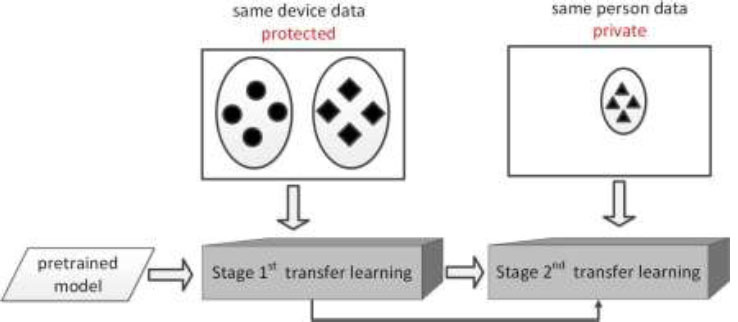
Overview of two-stage transfer learning.

**Fig. (2):**
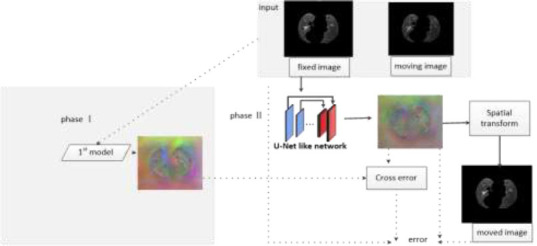
An overview of the method.

**Fig. (4):**
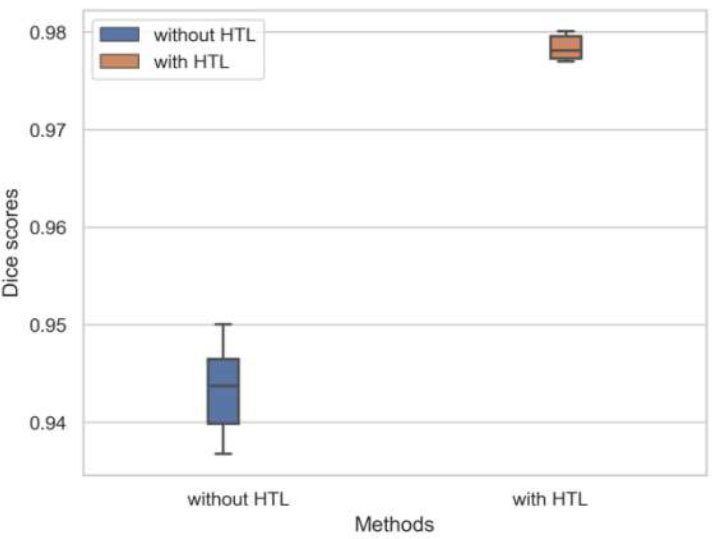
Results of the ablation study with hierarchical transfer learning.

**Fig. (5):**
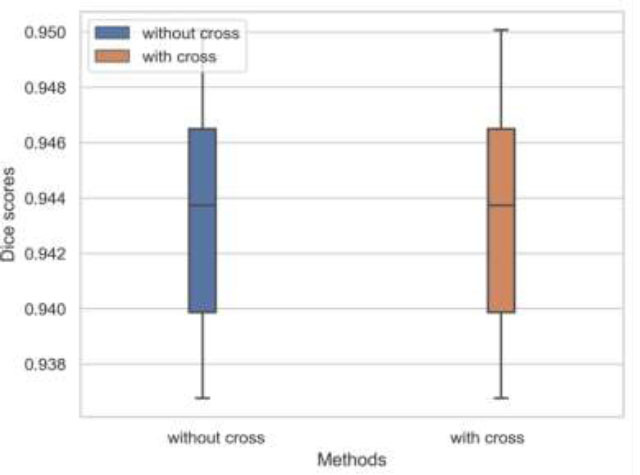
Ablation study with cross error.

**Fig. (6):**
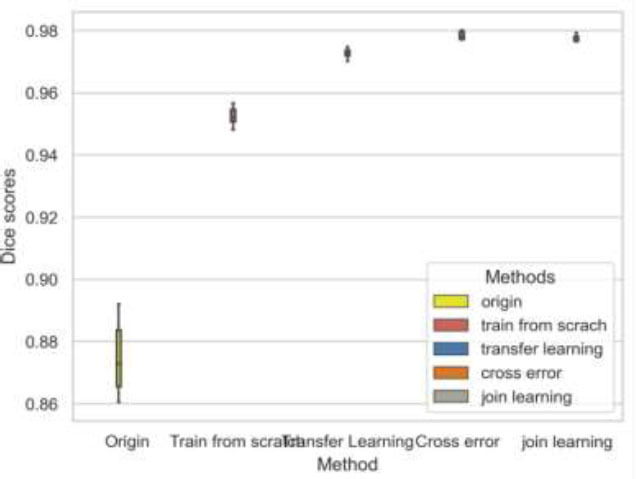
Comparison of training mode.

**Fig. (7):**
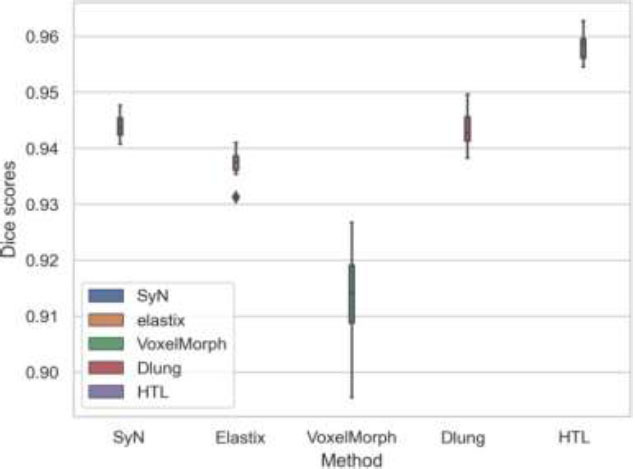
Comparison with the state-of-the-art method.

**Table 1:** Accuracy comparison.

Method	Avg Dice	Jacobian
SyN	0.91(0.002)	0(0)
Elastix	0.92(0.002)	1736(1106)
VoxelMorph	0.96(0.002)	17926
Dlung	0.962	31
Ours	0.978	11

## Data Availability

The data and supportive information is available within the article.

## LIST OF ABBREVIATIONS

HU: Hounsfield Unit
HTL: Hierarchical Transfer Learning
GPUs: Graphics Processing Units
